# CNN-Based Quality Assurance for Automatic Segmentation of Breast Cancer in Radiotherapy

**DOI:** 10.3389/fonc.2020.00524

**Published:** 2020-04-28

**Authors:** Xinyuan Chen, Kuo Men, Bo Chen, Yu Tang, Tao Zhang, Shulian Wang, Yexiong Li, Jianrong Dai

**Affiliations:** National Cancer Center/National Clinical Research Center for Cancer/Cancer Hospital, Chinese Academy of Medical Sciences and Peking Union Medical College, Beijing, China

**Keywords:** radiotherapy, quality assurance, automatic segmentation, deep learning, convolutional neural networks

## Abstract

**Purpose:** More and more automatic segmentation tools are being introduced in routine clinical practice. However, physicians need to spend a considerable amount of time in examining the generated contours slice by slice. This greatly reduces the benefit of the tool's automaticity. In order to overcome this shortcoming, we developed an automatic quality assurance (QA) method for automatic segmentation using convolutional neural networks (CNNs).

**Materials and Methods:** The study cohort comprised 680 patients with early-stage breast cancer who received whole breast radiation. The overall architecture of the automatic QA method for deep learning-based segmentation included the following two main parts: a segmentation CNN model and a QA network that was established based on ResNet-101. The inputs were from computed tomography, segmentation probability maps, and uncertainty maps. Two kinds of Dice similarity coefficient (DSC) outputs were tested. One predicted the DSC quality level of each slice ([0.95, 1] for “good,” [0.8, 0.95] for “medium,” and [0, 0.8] for “bad” quality), and the other predicted the DSC value of each slice directly. The performances of the method to predict the quality levels were evaluated with quantitative metrics: balanced accuracy, *F* score, and the area under the receiving operator characteristic curve (AUC). The mean absolute error (MAE) was used to evaluate the DSC value outputs.

**Results:** The proposed methods involved two types of output, both of which achieved promising accuracy in terms of predicting the quality level. For the good, medium, and bad quality level prediction, the balanced accuracy was 0.97, 0.94, and 0.89, respectively; the *F* score was 0.98, 0.91, and 0.81, respectively; and the AUC was 0.96, 0.93, and 0.88, respectively. For the DSC value prediction, the MAE was 0.06 ± 0.19. The prediction time was approximately 2 s per patient.

**Conclusions:** Our method could predict the segmentation quality automatically. It can provide useful information for physicians regarding further verification and revision of automatic contours. The integration of our method into current automatic segmentation pipelines can improve the efficiency of radiotherapy contouring.

## Introduction

Patients are scanned using computed tomography or magnetic resonance imaging at the start of radiotherapy. This generates the simulation images on which physicians manually contour the tumor target and organs at risk (OARs). The radiotherapy plan and the radiation dose are, respectively, designed and calculated as per these images.

Accurate contouring of the tumor target and OARs is a critical step in the development of effective radiotherapy plans because all subsequent steps in the planning of radiotherapy and treatment delivery process are dependent on these contours and are prerequisites for achieving the optimal curative effect for patients. The process of contouring is usually performed manually by physicians, making it time-consuming and subjective.

Automatic contouring can be a very useful tool in clinical practice to reduce the inter- and intra-observer variability and save time. With the application of deep learning methods in radiotherapy, automatic segmentation of tumor target and OARs becomes possible. The convolutional neural network (CNN) is a class of deep neural network that is usually applied to analyze visual imagery. The use of CNN (1, 2) in radiotherapy has been shown to be a state-of-the-art method for organs and tumor segmentation in several disease sites (3–11). Various notable developments in the use of deep learning methods for organ segmentation have improved the precision of automatic segmentation (12–15). However, considerable variability in medical images can cause unpredictable errors, even with the use of the best model. Hence, human interventions are still necessary. Physicians must spend a considerable amount of time examining and modifying the contours slice by slice. This greatly reduces the benefit of the segmentation tool's automaticity.

Physicians are interested in the accuracy of automatic segmentation generated by deep learning. The segmentation quality is traditionally evaluated on a separate test set using various metrics (16). These metrics reflect the agreement of predicted contours compared to the reference “ground truth.” However, the assessment of quality using traditional evaluation measures is not possible owing to the lack of the ground truth of the contour. Information about the quality of the contours generated by the segmentation model is lacking. Therefore, it is crucial to develop a method for evaluating the quality of segmentation and identifying the flawed contours. Although some studies (17, 18) have used statistical metrics derived from geometric distributions to determine the accuracy of contouring, these metrics are not always good indicators of organ contouring. Zhang et al. (19) proposed a texture-based method to validate the automatic contour propagation based on deformable image registration. This model exhibited good performance on two very complex organs. Regarding deep learning-based segmentation, some scholars have tried to predict the quality using “reverse testing,” (20, 21) whereby a new model is trained using the predictions of the test set and evaluated again on the training set. The reverse testing method requires many predicted results for training and cannot assess the quality of the automatic segmentation software output directly; therefore, it cannot provide individual evaluations in real time.

An increasing number of deep learning-based automatic segmentation tools is being introduced for routine clinical use. Although automatic segmentation is rapid, careful examination, and modification by the physicians are required. In this study, we proposed a fully automatic quality assurance (QA) method for deep learning-based segmentation with a CNN. We adopted a well-known CNN framework with high performance for quality prediction. To our knowledge, this is the first attempt of the application of a deep learning method in the field of radiotherapy for the QA of automatic segmentation. The proposed method can automatically assess the quality of the automatic segmentation. The promising results indicate a potentially wide application in the field of medicine. In combination with the automatic segmentation software, it can identify the regions of interest that need radiotherapy more quickly and accurately, thereby improving the efficiency of physicians.

## Materials and Methods

### Patient Data

The study cohort comprised 680 patients with early-stage breast cancer who were treated with breast-conserving lumpectomy and whole breast radiation at our hospital. These patients received adjuvant radiotherapy after lumpectomy. Our study only included patients who had received whole breast radiotherapy. Patients who had undergone axillary or supraclavicular radiotherapy were excluded. Thus, 340 patients had left-sided breast cancer, and the others had right-sided breast cancer. The CTV of breast cancer included most ipsilateral breast tissue. It was contoured on the CT image by one physician (with >5 years of working radiotherapy experience), rechecked, and approved by senior experts (with >10 years of working radiotherapy experience). All the physicians are credentialed radiation oncologists. Manual CTV delineations by experts were set as the ground truth of segmentation in this study. The CTV size on each 2D CT slice ranged from 440 to 12,440 pixels (4,510 ± 2,285).

The data for the planning CT were acquired using Somatom Definition AS 40 (Siemens Healthcare, Forchheim, Germany) or Brilliance CT Big Bore (Philips Healthcare, Best, the Netherlands) systems set on helical scan mode with voltage, tube current, and CIDI of 120 kVp, 150 mAs, and 10.96 mGy for Siemens CT and 120 kVp, 250 mAs, and 13.2 mGy for Siemens CT, respectively. CT images were reconstructed with a matrix size of 512 × 512 and a thickness of 5 mm. The pixel size was 0.98–1.27 (mean, 1.05) mm. CT images were resampled to an isotropic pixel resolution of 1.00 × 1.00 mm^2^. This protocol changed the size of the image matrix. We subsequently used cropping or zeroing to transform all the images into a uniform size of 512 × 512. A contrast-limited adaptive histogram equalization algorithm (22) was applied to enhance the image contrast.

We randomly selected the data from 520 cases (left: 260; right: 260) as the training dataset (to train the model), used 80 cases (left: 40; right: 40) for the validation set (to find the optimal model), and used the remaining 80 cases (left: 40, right: 40) as the test set (to assess the performance of the proposed method).

### Workflow of QA for Segmentation

**Figure 1:**
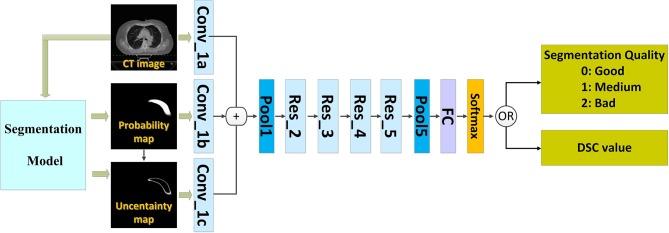
The architecture of the proposed method.

We introduced a QA workflow for segmentation with deep learning (Figure 1 illustrates the overall architecture). It had the following two main parts: a deep learning-based segmentation model and a QA network. The network of the segmentation model and that of the QA are independent of each other. The two networks were based on the CNN. The proposed pipeline included the following four main steps: (i) running the automatic segmentation method to obtain segmentation probability maps; (ii) calculating the uncertainty maps using the segmentation probability; (iii) predicting the segmentation quality using a classification model based on the CT images, probability maps, and uncertainty maps; and (iv) physician revision of the automatic segmentation according to their knowledge and the predicted quality.

We used the deep learning method (8) to build models for segmenting the CTVs of left- and right-sided breast cancers. We trained an automatic QA network with the data from both sides of breast cancer CTV to predict the quality of the segmentation models. Thus, it can be used for patients with breast cancer on either side.

#### Automatic Segmentation Network

Our study was not focused on the segmentation method. Therefore, we used an existing CNN (8) that had demonstrated high performance. Two-dimensional (2D) CT images were the inputs, while the corresponding 2D segmentation probability maps were the outputs. The “segmentation probability map” has the same resolution as the CT image. The value of each pixel represents the probability that the pixel belongs to the contour to be segmented. Most existing segmentation networks can achieve this. With this module, we can obtain contours that the segmentation model “thinks” is correct; however, we do not know the quality of the automatic segmentation before we have the ground truth of the contour.

#### The Inputs of QA Networks

The inputs of the network included the following three types of images: the 2D CT image, the generated probability map, and the uncertainty map.

The probability map (*p*) can be generated by the automatic segmentation model. Each pixel (*i, j*) denotes the probability that the pixel (*i, j*) belongs to the region to be segmented. The “probability map” represents the predicted contour to some extent. In addition, we added the 2D uncertainty map (*u*) to predict the segmentation quality. The uncertainty map is calculated as follows:

(1) $$\begin{array}{l} u(i,j)=\{\begin{array}{l} p(i,j),\;0\leq p(i,j)\leq 0.5\\ 1-p(i,j),\;0.5<p(i,j)<1\\ 0,\;p(i,j)=1 \end{array} \end{array}$$

where *u*(*i, j*) denotes the uncertainty of the pixel (*i, j*). The pixels with higher uncertainty correspond to the ones that lie close to the decision boundary of the segmentation model. Hence, the CNN model does not conclude how to segment such regions. Therefore, the uncertainty map is related closely to the quality of the automatic segmentation. Although the “uncertainty map” was calculated from the “probability map” using (Equation 1), we believe that both are necessary because the former map represents the confidence of the model. We intended to directly input these two parameters into the network to increase the speed and quality of learning.

In addition, the segmentation results are related to human anatomy and image, including geometric information and image contrast. Therefore, we also provide the CT image as an input to the network for the extraction of useful features.

#### The Outputs of QA Networks

Usually, the segmentation quality is quantified using the Dice similarity coefficient (DSC) (23) that measures the degree of overlap between the automated segmentation (A) and manual segmentation (B). In this study, the corresponding DSC of each slice is calculated as follows:

(2) $$\begin{array}{l} DSC(A,B)=\frac{2|A\cap B|}{\left|A|+|B\right|} \end{array}$$

where A represents the ground truth, B denotes the auto-segmented structure, and *A*⋂*B* is the intersection of A and B. DSC ranges from 0 (indicating no spatial overlap between the two segmentations) to 1 (indicating perfect concordance). We also define the DSC as 1 for the slice with no contour generated by the automatic segment method (B = 0) or by physicians (A = 0) because the performance of automatic segmentation is perfect.

We used the DSC as the index of segmentation quality. This study tests two types of DSC outputs. One predicts the quality levels for each slice, while the other predicts the DSC value for each slice directly. We divided the DSC range into three quality levels (0: good; 1: medium; 2: bad) based on our clinical experience and a review of the literature. The “good” level of DSC was set as [0.95, 1], the “medium” level as [0.8, 0.95], and the “bad” as [0, 0.8].

#### The Architecture of QA Networks

We applied convolutional architecture for fast feature embedding (Caffe) (24) on an NVIDIA TITAN X graphics card as the deep learning framework to implement the training and testing.

The CNN that can predict the segmentation quality is of a typical classification network. Theoretically, any CNN classification network can be applied in this study; however, we chose ResNet-101 (2) because of its excellent performance owing to its relation to image classification. The ResNet-101 classification network has five residual blocks (Figure 1). It has 101 convolutional layers and can extract low-level, middle-level, and high-level visual features. A batch-normalized option was used after each convolutional layer, followed by the application of a rectified-linear non-linearity (ReLU) max (0, x). Pooling was conducted in Res_3, Res_4, and Res_5 with a stride of two. After convolution and pooling operations, the output of Pool 5 had dimensions of 2,048 × 1 × 1. These final extracted features were utilized for quality classification prediction. A fully connected layer reduced the output dimensions to 3 × 1 × 1. Subsequently, a softmax layer classified the segmentation quality into the following three categories: good, medium, and bad.

In order to keep the details of human tissues, the input image had a larger size of 512 × 512 when we performed the segmentation. For predicting the segmentation quality, we down-sampled all the input images to 224 × 224 pixels to fit the requirement of the adopted ResNet-101 as well as to make the model small and fast.

During the training phase, we adopted random left–right flipping, cutting, and rotation (between 5 and 5 degrees) for data augmentation (25). This expanded the existing training dataset and avoided network over fitting. We oversampled the cases of the minority class (i.e., class with fewer training cases) to solve the imbalance problem (26) (where the classes were unequal). To accelerate training, the quality prediction networks were trained with the initial parameters from the ResNet-101 model trained on ImageNet and were then fine-tuned using the data of the breast cancer CTV. No parameter of the model was frozen during the fine-tuning. The network was trained using a batch size of 16, momentum of 0.9, weight decay of 0.0005, learning rate policy of poly, initial learning rate of 0.00025, and power of 0.9. The training was stopped after 100 K iterations.

### Quantitative Analysis of Prediction Accuracy

The performance of the QA model regarding quality levels prediction was evaluated using quantitative metrics (27) that included the balanced accuracy (BA), *F* score, and the area under the receiving operator characteristic (ROC) curve (AUC). They were defined as follows:

(3) $$\begin{array}{l} BA=\frac{1}{2}(\frac{TP}{TP+FN}+\frac{TN}{TN+FP}) \end{array}$$

(4) $$\begin{array}{l} F\;\text{score}=\frac{2TP}{2TP+FN+FP} \end{array}$$

where TP is the number of true-positives, FP is the number of false-positives, TN is the number of true-negatives, and FN is the number of false-negatives. BA and *F* score are usually chosen for data with only binary output; however, our experiment had the following three classes: good, medium, and bad. Here, we computed the TP, TN, FP, and FN for every class *I*∈*G* = {*good, medium, bad*}, such that the *i*th matrix considered class *g*_*i*_ as the positive class and all other classes *g*_*j*_ with *j* ≠ *i* as the negative class. In the “good” class, for example, TP meant “good” contour was correctly identified as “good,” FP meant “good” contour was incorrectly identified (classified as “medium” or “bad”), TN meant “medium” or “bad” contour was correctly rejected as “good,” and FN meant “medium” or “bad” contour was incorrectly rejected (classified as “good”). We also performed the experiments using different combinations of the three inputs (CT image, probability map, or uncertainty map) for comparison.

The performance of the QA model regarding DSC values prediction was evaluated using mean absolute error (MAE). It is defined as shown in Equation (5).

(5) $$\begin{array}{l} MAE=\frac{1}{N}\sum_{i=1}^{N}\left|DSC_{pred}(i)-DSC_{GT}(i)\right| \end{array}$$

Where *i* is the index of the slice of each patient in the test set, *N* is the total number of slices for all patients in the test set, and *DSC*_*pred*_ and *DSC*_*GT*_ are the predict and ground truth of DSC, respectively.

## Results

### Prediction Accuracy of the Quality Levels

Table 1 lists the quality prediction results of the test set. The CNN model with CT image, probability map, and uncertainty map demonstrated the best performance with AUC values of 0.96, 0.93, and, 0.88 for “good,” “medium,” and “bad” quality, respectively. The BA of all the quality levels was >0.89, indicating that >89% of automatic segmentation could be classified accurately into a quality level. The performance was inferior with only one of the three labels. This comparative experiment indicated that including all three labels as input produced the optimal quality level prediction model. The model only with the CT label performed the worst for all quality levels. The performances of the two models were comparable only when the uncertainty or probability label was used. When either the probability or the uncertainty map was removed from the input, the results of the models were very close to those obtained using all the three inputs, although the performance was slightly worse. This result may be due to the generation of the uncertainty map from the probability map, such that additional information was not added to the model.

**Table 1:** Results of quality prediction.

**Model input**	**GT**	**BA**	***F* score**	**AUC**
All three labels	Good	0.97	0.98	0.96
	Medium	0.94	0.91	0.93
	Bad	0.89	0.81	0.88
CT image	Good	0.92	0.92	0.91
	Medium	0.89	0.87	0.88
	Bad	0.83	0.76	0.80
Probability map	Good	0.96	0.97	0.95
	Medium	0.93	0.91	0.93
	Bad	0.87	0.80	0.86
Uncertainty map	Good	0.96	0.97	0.95
	Medium	0.93	0.91	0.93
	Bad	0.87	0.80	0.86
CT image + probability map	Good	0.97	0.98	0.96
	Medium	0.93	0.91	0.93
	Bad	0.88	0.80	0.87
CT image + uncertainty map	Good	0.97	0.97	0.96
	Medium	0.93	0.91	0.93
	Bad	0.89	0.81	0.88

**Figure 2:**
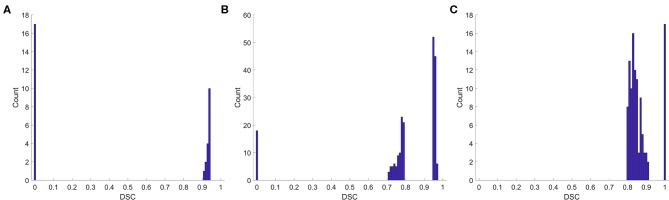
Histogram of the DSC for incorrect prediction. **(A)** Misclassified as “good,” **(B)** misclassified as “medium,” **(C)** misclassified as “bad”.

We also analyzed the DSC for incorrect prediction. Figure 2 shows the distribution. Nearly half of the contours that were misclassified as “good” quality (Figure 2A) had a DSC of ~0.95, the boundary between “good” and “medium.” Most contours misclassified as “medium” quality (Figure 2B) were also at the boundary of the quality classification. However, the contours misclassified as “bad” quality (Figure 2C) had a wider DSC range. In addition, few contours with a DSC of 0 or 1 were not identified correctly. A classification of “0” meant that the segmentation model missed or generated redundant contours, whereas “1” meant that no contour was generated by the segmentation model or by physicians. These slices were usually located at the top or bottom boundaries of the CTV, where the classification model could be inaccurate.

**Figure 3:**
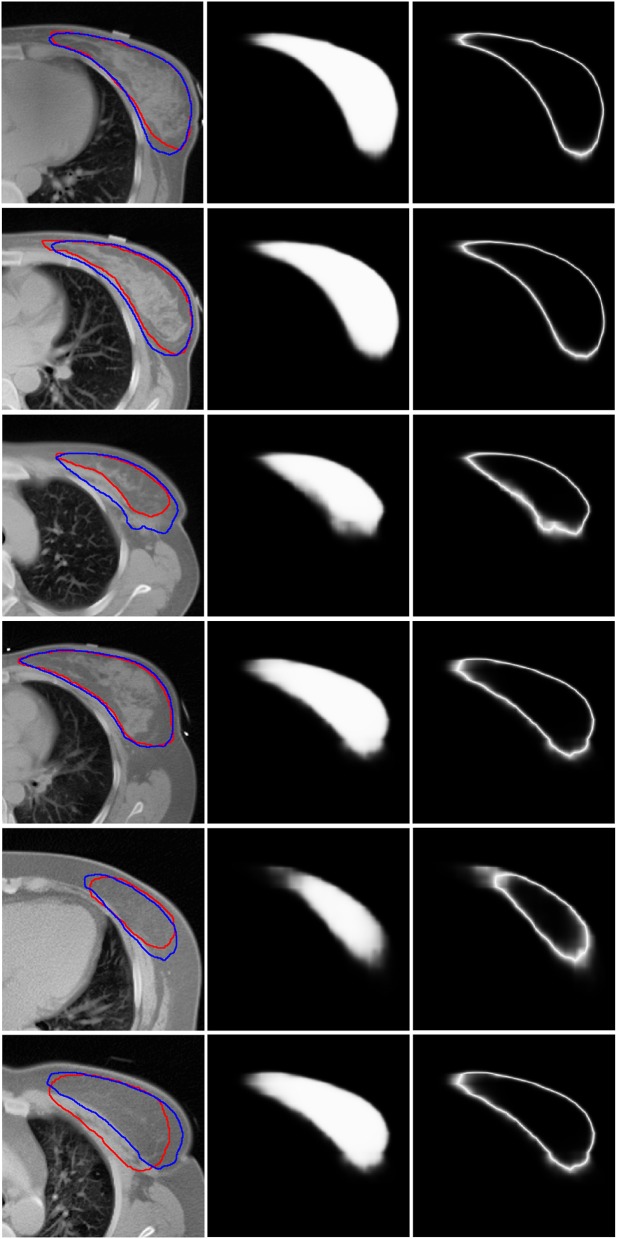
Six representative examples of accurate and inaccurate predictions. The first column shows the CT images with ground truth (Red line) and auto-segmentation (Blue line). The second column shows the probability maps. The third column shows the uncertainty maps. Rows 1–3 show the contours of the “good,” “medium,” and “bad” levels that had been classified accurately. Rows 4–6 show the contours of the “good,” “medium,” and “bad” levels that were misclassified as “medium,” “bad,” and “medium,” respectively.

Figure 3 shows six representative examples of accurate and inaccurate predictions. The first column shows the CT images with the ground truth (Red line) and auto-segmentation (Blue line); the second column shows the probability maps; and the third column shows the uncertainty maps. Rows 1–3 show the contours of the “good,” “medium,” and “bad” levels that were classified correctly, respectively, while rows 4–6 show the contours of the “good,” “medium,” and “bad” levels that were misclassified as “medium,” “bad,” and “medium,” respectively.

### Prediction Accuracy of the DSC Value

**Figure 4:**
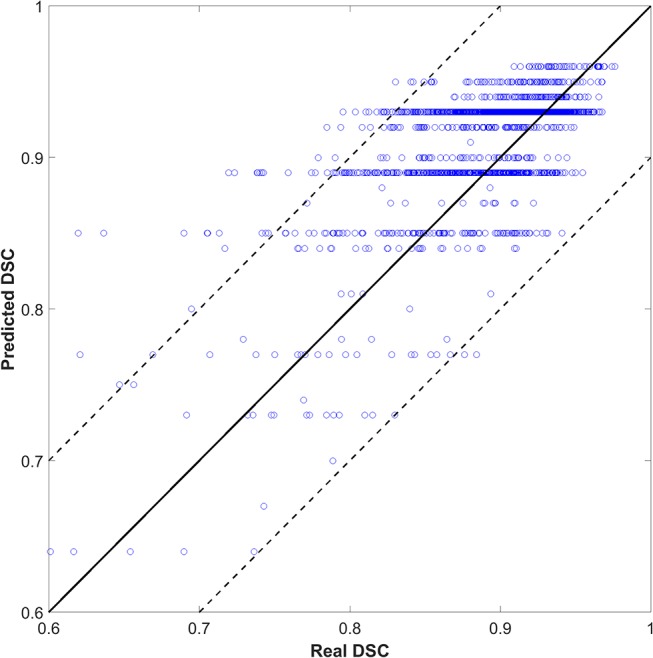
The result of DSC prediction.

We also tried to predict the DSC directly instead of the quality levels of “good,” “medium,” or “bad,” as a discretized version of the DSC. The output (DSC) was set to 101 classes (i.e., 0 to 100 with an interval of 1). Figure 4 shows the scatter plots of predicted vs. real DSC values for all the test contours. The two dashed lines represent a margin of error of ±10% relative to the true value. The points should form a diagonal line if the prediction is good. The predicted DSC value was close to its real value, with a mean absolute error of 0.06 ± 0.19. The correlation coefficient was 0.75. Specifically, 80, 85, and 93% of the prediction had an absolute error within 0.03, 0.05, and 0.10, respectively, compared to the reality. There was no significant correlation between the prediction error and CTV size that had a correlation coefficient of 0.05.

As shown in Figure 4, the predicted DSC values were discretized to <101 levels. Possibly, because the tasks were divided into 101 categories, there were insufficient training samples for each category. As such, the model could not yield accurate predictions. We continue to accumulate samples and hope to improve this effect.

### Prediction Time

We tested the time cost for quality prediction using an NVIDIA TITAN X graphics card. The speed was fast with a mean time of 2 s per patient, while a physician took about 10 min to review the CTV.

## Discussion

We proposed a QA process for automatic segmentation. A CNN-based method can predict the segmentation quality automatically. Our QA method can be used to efficiently differentiate between high-quality and low-quality contours. These low-quality contours can be automatically selected for physicians in order to perform further verification and revision. This can improve the efficiency of the existing automatic contouring process.

We adopted a well-known network (ResNet-101) in our method. The network structure is not novel in the traditional computer vision community. However, the application of using an old method for a new problem is also recognized as innovative. To our knowledge, this is the first attempt at applying this method in the field of radiotherapy for the QA of auto-segmentation. The promising results indicate a potentially wide application in medicine. The proposed method is novel and has four main contributions. First, the proposed method can predict, in real time, the performance of a segmentation model on each individual slice to help physicians review the contours. Second, maps of segmentation probability and uncertainty were introduced to predict the contours' quality. These two kinds of maps can directly reflect the confidence of the segmentation model that is closely related to its performance. Third, the proposed method can predict the segmentation quality slice by slice based on the DSC that can provide a quantitative index for the physicians to use their judgment. Finally, the proposed method can be integrated into the current segmentation pipelines in clinical practice to improve efficiency.

We also investigated the influence of each channel. Our comparison showed that using only one of the three labels (CT image, probability map, or uncertainty map) led to degradation of the prediction performance. When we used “CT image + probability map” or “CT image + uncertainty map” as inputs, the results of the models were very similar to but slightly worse than that obtained using all three inputs. This shows that the CNN can extract useful information from the three types of labels and improve the robustness and accuracy of the model. In this study, the uncertainty map was generated from the probability map of the automatic segmentation model; the performance of the two models was comparable only when uncertainty or probability was used as the input. The “probability map” reflects the segmentation result, and the “uncertainty map” represents the confidence of the model. Both the maps should be useful for predicting the contour quality. The model using only CT demonstrated the worst performance that was expected because CT images do not contain contour information for automatic segmentation and cannot accurately evaluate the contour quality.

We selected the threshold for good, medium, and bad segmented quality levels according to our experience and previous reports. Several studies have shown the existence of some interobserver variability in the clinical target for breast cancer, with the corresponding DSC results ranging from 0.88 to 0.93 (8, 28–31). As such, based on published literatures and our clinical experience, the “good” level range of DSC was set ≥0.95 in this study. A good level of slices means that the radiation oncologists do not need to perform many changes of automatic contouring. It is widely considered that DSC ≥ 0.7 is an acceptable consistency (32, 33). In order to reduce the DSC bias of the large target, we set a DSC of 0.8 as the boundary of “bad” level that suggested manual contouring. The “medium” level means that the radiation oncologist needs to make moderate modifications. Regardless of the threshold that is used, we believe that the misclassification will always be close to the boundary. We will explore more reasonable threshold determination methods in the future.

This study also tested the methods of predicting DSC values directly. However, the accuracy of prediction may decrease with an increase in the classification types. This low accuracy may be a result of small differences in the characteristics between samples with similar DSC that made it difficult for the classifier to make an accurate prediction.

This method potentially plays an important role in online adaptive radiotherapy (ART); automatic contouring is a very critical step here. It is necessary to manually review the automatically generated contours slice by slice although the automatic segmentation tools can reduce time. This is a big challenge for ART because it increases a physician's workload and lowers the patient's comfort. The proposed method highlights the low-quality contours for manual review, thereby improving the efficiency of ART.

There are three main limitations of this preliminary study. First, we used the CTV of breast cancer to demonstrate the proposed QA method; however, we did not investigate the CTVs for other types of cancers and OARs. The potential for reducing the time required to evaluate contours improves further if the method is applied to both OARs and CTV because a single case always has many OARs. In addition, the segmentation of a CTV of breast cancer is relatively easy because this tumor type is known to exhibit good contrast with the surrounding tissues. The auto-segmentation methods achieved very good performances on this structure, indicating that the proposed QA model is reliable. However, for other tumor sites or even OARs, the auto-segmentation model would demonstrate poorer performance or even fail. Future research should focus on broader experiments across all treatment sites, as well as on OARs. Second, DSC is sensitive to the absolute volume of the contour. For a small-volume contour, a low DSC value does not necessarily indicate an inaccurate contour. We only used the general DSC for modeling that may have caused mislabeling of the contour slices. The Hausdorff distance or surface DSC (6) is a possible alternative metric for evaluating the segmentation performance, and this can be adopted in our newly developed segmentation QA networks. Third, a small uncertainty means that the segmentation model is more confident that the pixel does or does not belong to the CTV. However, the uncertainty may not be related directly to the performance of the segmentation model. Network confidence about a prediction does not necessarily correlate with an accurate prediction. Rather, network confidence in an area with systematic errors from the model may be attributable to the unrepresentative training datasets or intra- and inter-observer variation.

In our future potential research, the QA method will be incorporated into the automatic segmentation process. Segmentation can benefit from a quality-assurance-in-the-loop workflow that can be used to predict the quality for the segmentation model to “tune” the parameters further. In addition, the proposed segmentation QA method can be adopted in radiotherapy and in other fields of segmentation, including radiology.

## Conclusions

The QA of automatic segmentation is an important step in radiotherapy. We used a convolutional network for the QA of automatic segmentation in this study. The network can learn the useful features from the three types of images, classify the contouring into different quality levels, or predict the DSC value directly with both high efficiency and high speed. The proposed method can provide useful information for physicians for rapid verification and revision. The performance can be improved if such information can be integrated into the computer-assisted automatic segmentation system.

## Data Availability Statement

The datasets generated and/or analyzed during the current study are not publicly available due to data security requirement of our hospital. Requests to accessed the data should be sent to Kuo Men/ menkuo@icams.ac.cn.

## Ethics Statement

The studies involving human participants were reviewed and approved by the Independent Ethics Committee of Cancer Hospital, Chinese Academy of Medical Sciences. Written informed consent for participation was not required for this study in accordance with the national legislation and the institutional requirements.

## Author Contributions

XC and KM wrote the programs, performed data analysis, and drafted the manuscript. TZ helped to check the contours. BC and YT analyzed and interpreted the patients' data. SW, YL, and JD guided the study and participated in discussions and preparation of the manuscript. All authors read, discussed, approved the final manuscript, and conceived the study design.

## Conflict of Interest

The authors declare that the research was conducted in the absence of any commercial or financial relationships that could be construed as a potential conflict of interest.

## Abbreviations

CNN: convolutional neural networks
CTV: clinical target volume
AUC: area under the receiving operator characteristic curve
OARs: organs at risk
GT: ground truth
CT: computed tomography
DSC: Dice similarity coefficient
BA: balanced accuracy
Caffe: convolutional architecture for fast feature embedding
ART: adaptive radiotherapy.
